# Anti-T cell immunoglobulin and mucin domain-2 monoclonal antibody exacerbates collagen-induced arthritis by stimulating B cells

**DOI:** 10.1186/ar3288

**Published:** 2011-03-22

**Authors:** Toshio Kawamoto, Yoshiyuki Abe, Jun Ito, Fumihiko Makino, Yuko Kojima, Yoshihiko Usui, Juan Ma, Shinji Morimoto, Hideo Yagita, Ko Okumura, Yoshinari Takasaki, Hisaya Akiba

**Affiliations:** 1 Department of Immunology, Juntendo University School of Medicine, 2-1-1 Hongo, Bunkyo-ku, Tokyo 113-8421, Japan; 2 Department of Internal Medicine and Rheumatology, Juntendo University School of Medicine, 2-1-1 Hongo, Bunkyo-ku, Tokyo 113-8421, Japan; 3 Department of Respiratory Medicine, Juntendo University School of Medicine, 2-1-1 Hongo, Bunkyo-ku, Tokyo 113-8421, Japan; 4 Division of Biomedical Imaging Research, Juntendo University School of Medicine, 2-1-1 Hongo, Bunkyo-ku, Tokyo 113-8421, Japan; 5 Department of Ophthalmology, Tokyo Medical University, 6-7-1 Nishi-shinjuku, Shinjuku-ku, Tokyo 160-0023, Japan

## Abstract

**Introduction:**

T cell immunoglobulin and mucin domain-2 (TIM-2) has been shown to regulate CD4 T cell activation. However, the role of TIM-2 in the autoimmune disease models has not been clarified yet. In this study, we investigated the effects of anti-TIM-2 monoclonal antibodies (mAbs) in collagen-induced arthritis (CIA) to determine whether TIM-2 contributes to the development of T helper (Th) 1 or Th17 cells and joint inflammation.

**Methods:**

DBA/1 mice were treated with anti-TIM-2 mAbs during the early or late phase of CIA. Type II collagen (CII)-specific CD4 T-cell proliferative response and cytokine production were assessed from lymph node cell culture. The serum levels of CII-specific antibody were measured by ELISA. The expression of TIM-2 on CD4 T cells or B cells was determined by flow cytometric analysis.

**Results:**

Administration of anti-TIM-2 mAbs in early phase, but not late phase, significantly exacerbated the development of CIA. Although anti-TIM-2 mAbs treatment did not affect the development of Th1 or Th17 cells in the draining lymph node, the serum levels of anti-CII antibodies were significantly increased in the anti-TIM-2-treated mice. TIM-2 expression was found on splenic B cells and further up-regulated by anti-immunoglobulin (Ig)M, anti-CD40, and interleukin(IL)-4 stimulation. In contrast, CD4 T cells did not express TIM-2 even when stimulated with both anti-CD3 and anti-CD28 mAbs. Interestingly, anti-TIM-2 mAbs enhanced proliferation and antibody production of activated B cells in vitro.

**Conclusions:**

TIM-2 signaling influences both proliferation and antibody production of B cells during the early phase of CIA, but not induction of Th1 or Th17 cells.

## Introduction

The T cell immunoglobulin and mucin domain (TIM) family has recently been implicated in the regulation of T cell activation and immune responses [1,2]. The genes of this family were found within the *Tapr *(T cell and airway phenotype regulator) locus on mouse chromosome 11B1.1, which is syntenic to human chromosome 5q33.2, a region that has been linked to allergic diseases [3]. To date, four proteins (TIM-1, -2, -3, and -4) have been identified in mice and three proteins (TIM-1, -3, and -4) have been found in humans [2]. In the mouse, TIM-1 and TIM-3 have polymorphism at the protein level, represented by BALB/c-type and B6-type [3]. No human orthologue for mouse TIM-2 has been identified although, given its close sequence homology, TIM-1 may share some of its functions [1-5]. All proteins are type I transmembrane proteins with common structural motifs including extracellular IgV and mucin domains, and intracellular domains. TIM-1, TIM-2, and TIM-3, but not TIM-4, contain a conserved intracellular tyrosine phosphorylation motif that is involved in transmembrane signaling [2,3].

TIM-2 was also identified as a ligand for semaphoring 4A (Sema4A), which was expressed on activated macrophages, B cells, and dendritic cells [6]. Further study has identified another ligand for TIM-2, the heavy chain of ferritin (H-ferritin) [4]. Ferritin is a major tissue iron-binding protein, which is composed of heavy and light chain subunits [7]. Expression of TIM-2 has been found on B cells, epithelial cells in the liver and kidney, and oligodendrocytes, although the function of TIM-2 in these cells has not yet been understood [4,8]. It has also been reported that TIM-2 is preferentially expressed on T helper (Th) 2 cells [9,10]. Some studies support roles for TIM-2 as a negative regulator of Th2 immune responses [5,9,10]. Initial studies showed that soluble TIM-2-Ig fusion protein induced T cell hyperproliferation and enhanced production of Th2 cytokines *in vivo *[9]. A subsequent study also showed that TIM-2-Ig treatment exacerbated lung inflammation in the ovalbumin (OVA)-induced asthma model [10]. Eosinophil numbers were increased in bronchial lavage, lymph node (LN) cell proliferation in response to OVA was increased, and production of Th2-type cytokines was heightened. Moreover, TIM-2-deficient mice showed an exacerbated lung inflammation in the OVA-induced asthma model [10]. Thus, these findings have suggested that TIM-2 is a critical negative regulator of Th2 immune responses. However, the immunological function of TIM-2 under Th1-polarizing conditions has not been investigated extensively. Here, we have examined the function of TIM-2 in the development of collagen-induced arthritis (CIA), which is a Th1-mediated autoimmune disease model, by administering a newly generated anti-TIM-2 monoclonal antibodies (mAbs). Our present results suggest that TIM-2 signaling influences both proliferation and antibody production of B cells during the early phase of CIA, but not induction of Th1 or Th17 cells.

## Materials and methods

### Animals and cells

Male DBA/1 mice and female Sprague Dawley rats were purchased from Charles River Laboratories (Kanagawa, Japan). FcRγ-deficient mice were supplied by Y. Suzuki (Department of Nephrology, Juntendo University, Tokyo, Japan) [11,12]. All mice were 7 to 10 weeks old at the start of experiments and kept under specific pathogen-free conditions during the experiments. All animal experiments were approved by Juntendo University Animal Experimental Ethics Committee. A cDNA fragment encoding the entire open reading frame of mouse TIM-2 molecule was prepared by RT-PCR from Con A-activated splenocytes of C57BL/6 mouse. The PCR product was cloned into pMKITneo vector and transfected into NRK-52E (normal rat kidney) or L5178Y (murine T lymphoma) cells by electroporation (TIM-2/NRK or TIM-2/L5178Y). Stable NRK-52E cells expressing TIM-1-BALB, TIM-1-B6, TIM-3-BALB, TIM-3-B6, or TIM-4 were also established in our laboratory by similar methods. These cells were cultured in RPMI1640 medium containing 10% FCS, 10 mM HEPES, 2 mM L-glutamine, 0.1 mg/ml penicillin and streptomycin, and 50 μM 2-ME.

### Generation of anti-mouse TIM-2 mAbs

The anti-mouse TIM-2 mAbs were generated by immunizing Sprague Dawley rats with TIM-2-Ig, consisting of the extracellular domain (aa 1-230 of mouse TIM-2) [3] and the Fc portion of mouse IgG2a, emulsified in complete Freund's adjuvant (CFA: Difco Laboratories, Detroit, MI, USA). Three days after the final immunization, LN cells were fused with P3U1 myeloma cells. After hypoxanthine-aminopterin-thymidine selection, hybridomas producing anti-TIM-2 mAb (RMT2-14, rat IgG2a/λ; RMT2-25, rat IgG2a/κ; and RMT2-26, rat IgG2b/κ) were selected by their reactivity to mouse TIM-2-transfected cells, but not to parental cells, by flow cytometry and then cloned by limiting dilution. The mAbs were purified from ascites of SCID mice by the octanoic acid and ammonium sulfate precipitation method, and purity was verified by SDS-PAGE analysis. Anti-TIM-1 (RMT1-17), anti-TIM-3 (RMT3-23), and anti-TIM-4 (RMT4-53) mAbs were also generated previously [13,14].

### Induction of CIA, mAb treatment, and clinical assessment of arthritis

DBA/1 mice (10 mice per group) were immunized intradermally at the base of the tail with 200 μg of bovine type II collagen (CII; Collagen Research Center, Tokyo, Japan) in 0.05 M acetic acid, emulsified in CFA. Twenty-one days after primary immunization, some groups of mice were boosted in the same way with 200 μg of CII in 0.05 M acetic acid, emulsified in incomplete Freund's adjuvant (IFA). The immunized mice were randomly selected and intraperitoneally administered with 300 μg of anti-TIM-2 mAbs or control rat IgG (Sigma-Aldrich, St Louis, MO, USA) every three days from day 0 to day 42, or day 0, 2, 5, 8, 11, 14, and 17 for the early phase, or day 15, 17, 20, 23, 26, 29, and 32 for the late phase. Mice were monitored for arthritis every day and scored in a blinded manner. The swelling of four paws was graded from 0 to 4 as follows: grade 0, no swelling; grade 1, one inflamed digit; grade 2, two inflamed digits; grade 3, more than one digit and footpad inflamed; and grade 4, all digits and footpad inflamed. Each paw was graded, and the four scores were totaled so that the maximal score per mouse was 16. Incidence was expressed as the percentage of mice that showed paw swelling in the total number of mice examined.

### Histological analysis

CIA mice were killed at day 45. The hind limbs were removed and fixed in buffered formalin, decalcified in 5% methyl alcohol and 5% formic acid, embedded in paraffin, sectioned, and stained with H&E.

### T cell stimulation *in vitro*

Draining LN cells from 10 mice were isolated and pooled in each group, and triplicate cultured in 96-well flat-bottom microculture plates at a density of 6 × 10^5 ^cells/well in the presence or absence of the indicated doses of denatured CII (dCII: 60°C, 30 minutes). In another experiment, DBA/1 mice (5 mice per group) were immunized subcutaneously with 5 μg of OVA with CFA and intraperitoneally administrated with 300 μg of RMT2-14 or control rat IgG on days 0, 2, and 4. On day 7, LN cells (6 × 10^5 ^cells/well) from five mice were pooled in each group and restimulated with the indicated doses of OVA in 96-well plates. All cultures were pulsed with ^3^H-thymidine (0.5 μCi/well; PerkinElmer, Waltham, MA, USA) for the last six hours of a 72-hour or 96-hour culture and harvested on a Micro 96 Harvester (Molecular Devices, Sunnyvale, CA USA). Incorporated radioactivity was measured on a microplate beta counter (Micro β Plus; PerkinElmer, Waltham, MA, USA). To determine the production of cytokines, cell-free supernatants were collected from each well at 72 hours or 120 hours and assayed for IFN-γ or IL-17 using Mouse IFN-γ or IL-17 ELISA Ready-SET-Go! kit (eBioscience, San Diego, CA, USA) according to the manufacturer's instructions.

### Preparation of activated CD4 and B cells

CD4 T cells were purified from the spleen of DBA/1 mice by passing it through a nylon wool column (Wako Pure Chemical Industries, Osaka, Japan) and by using an auto-MACS columns with CD4 T cell isolation kit (Miltenyi Biotec, Bergisch Gladbach, Germany) according to the manufacturer's instructions. Purified CD4 T cells were stimulated with immobilized anti-CD3 mAb (5 μg/ml) in the presence or absence of anti-CD28 mAb (5 μg/ml). B cells were also purified by using the auto-MACS column with B cell isolation kit. Purified B cells were stimulated with anti-IgM antibody (Ab) (5 μg/ml), anti-CD40 mAb (5 μg/ml) and/or recombinant mouse IL-4 (20 ng/ml) for 48 hours. The anti-CD3 (145-2C11), anti-CD40 (HM40-3), and recombinant mouse IL-4 were purchased from eBioscience (San Diego, CA, USA). Goat anti-mouse IgM F(ab')2 Ab was purchased from Jackson ImmunoResearch Laboratories (West Grove, PA, USA). Anti-CD28 (PV-1) mAb was kindly provided by Dr. R. Abe (Tokyo University of Science, Chiba, Japan) and Dr. C. June (University of Pennsylvania, Philadelphia, PA, USA).

### Flow cytometric analysis

Cells (0.5 to 1 × 10^6^) were first preincubated with unlabeled anti-CD16/32 mAb to avoid non-specific binding of Abs to FcγR and then incubated with biotinylated mAbs. After washing with PBS twice, the cells were incubated with PE-labeled streptavidin. After washing with PBS twice, the stained cells (live-gated on the basis of forward and side scatter profiles and propidium iodide exclusion) were analyzed on a FACSCalibur (BD Biosciences, San Jose, CA, USA), and data were processed using the CellQuest program (BD Biosciences, San Jose, CA, USA). Purified anti-CD16/32 (2.4G2) was purchased from BD Biosciences (San Jose, CA, USA). FITC-conjugated anti-CD3 (145-2C11) and CD19 (MB19-1), allophycocyanin-conjugated anti-CD4 (RM4-5) and CD45R/B220 (RA3-6B2), rat IgG isotype control, and PE-labeled streptavidin were purchased from eBioscience (San Diego, CA, USA).

### Serum anti-CII antibody levels

Sera were collected from each mouse on day 16, 24, or 32 and the titers of anti-CII IgG Abs were measured by ELISA. Bovine CII (1 μg/ml) was coated onto 96-well ELISA plates overnight at 4°C. After blocking with 1% BSA in PBS, serially diluted serum samples were added and incubated for one hour at room temperature. After washing, biotin-conjugated rat anti-mouse IgG1, IgG2a, or IgG2b mAbs (BD Biosciences, San Jose, CA, USA) were added and incubated for one hour at 37°C, washed, and then developed with Vectastain ABC kit (Vector Laboratories, Burlingame, CA, USA) and o-phenylendiamine (Wako Pure Chemical Industries, Osaka, Japan). After terminating the reaction with 2N H_2_SO_4_, OD at 490/595 nm was measured on a microplate reader (Bio-Rad, Hercules, CA, USA). A standard serum composed of a mixture of sera from arthritic mice was added to each plate in serial dilutions and a standard curve was constructed. The standard serum was defined as one unit and the antibody titers of serum samples were determined by the standard curve.

### *In vitro *B cell proliferation and Ig production assays

Purified B cells (1 × 10^5^/well) from DBA/1, BALB/c, or FcRγ-deficient mice were triplicate cultured with anti-IgM Ab (5 μg/ml), anti-CD40 mAb (5 μg/ml), and/or recombinant mouse IL-4 (20 ng/ml) in the absence or presence of human H-ferritin (EMD Chemicals, Gibbstown, HJ, USA) in 96-well flat-bottomed plates. Anti-CD16/32 mAb (5 μg/ml) and 10 μg/ml of anti-TIM-2 mAbs or control rat IgG were also added at the start of culture. To assess proliferative responses, the cultures were pulsed with ^3^H-thymidine (0.5 μCi/well) for the last six hours of a 72-hour culture and harvested. Incorporated radioactivity was measured as described above. For analysis of Ig secretion, 50 μl of day 7 culture supernatants were subjected to the cytometric bead array (CBA) using Mouse Immunoglobulin Isotyping Kit (BD Biosciences, San Jose, CA, USA) according to the manufacturer's instructions. This kit is highly sensitive and useful for a qualitative assay, but not a quantitative assay.

### Statistical analysis

Statistical analyses for parametric data were performed by unpaired Student's *t*-test. Nonparametric data were analyzed by the Mann-Whitney *U *test. Incidence was analyzed by Logrank test. The results are expressed as the mean ± standard error of the mean. Values of *P *< 0.05 were considered significant.

## Results

### Establishment of anti-mouse TIM-2 mAbs

**Figure 1:**
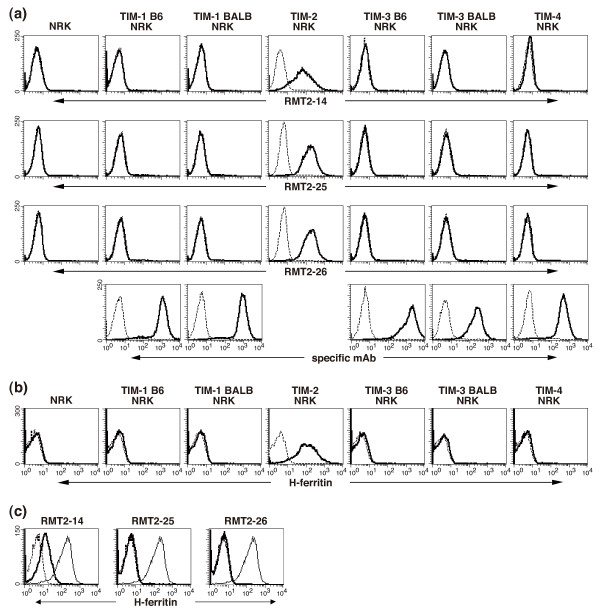
**Characterization of anti-TIM-2 mAbs**. **(a) **Reactivity of anti-T cell immunoglobulin and mucin domain (TIM)-2 monoclonal antibodies (mAbs) to mouse TIM family molecules. NRK-52E-derived TIM transfectants and parental cells were stained with biotinylated RMT2-14, RMT2-25, RMT2-26, specific mAbs against each TIM family molecule, or control IgG followed by PE-labeled streptavidin. Thick lines indicate the staining with the respective mAb and the dotted lines indicate background staining with control IgG. **(b) **H-ferritin binds to TIM-2 transfectant. NRK-52E-derived TIM transfectants were stained with Alexa647-labeled H-ferritin. Thick lines indicate the staining with the H-ferritin and the dotted lines indicate background staining with PBS. **(c) **Anti-TIM-2 mAbs inhibit H-ferritin binding to TIM-2 transfectant. TIM-2/L5178Y cells were pretreated with the indicated anti-TIM-2 mAb (thick lines) or control IgG (solid lines) and then stained with Alexa647-labeled H-ferritin. The dotted lines indicate background staining with PBS.

We immunized Sprague Dawley rats with TIM-2-Ig chimera protein and screened the hybridomas producing mAb that reacted with TIM-2 transfectants but not parental cells. Three mAbs, designated RMT2-14, RMT2-25, and RMT2-26 were selected. As shown in Figure 1a, all these mAbs reacted with TIM-2/NRK cells but not with parental NRK or the other TIM family-transfected (TIM-1 B6/NRK, TIM-1 BALB/NRK, TIM-3 B6/NRK, TIM-3 BALB/NRK, and TIM-4/NRK) cells. To characterize the antigen recognized by these mAbs, cell lysates of TIM-2/L5178Y or L5178Y cells were immunoprecipitated with these mAbs. Then the precipitates were analyzed by SDS-PAGE under nonreducing conditions and immunoblotting with biotin-conjugated RMT2-14, RMT2-25, or RMT2-26. All three mAbs precipitated an approximately 55 kDa protein from TIM-2/L5178Y cells, but not from L5178Y cells, consistent with the molecular mass of TIM-2 previously reported [6] [See Additional file 1]. To further determine whether these mAbs bind to the same epitope in the TIM-2 molecule, TIM-2/L5178Y cells were pre-incubated with unlabeled mAbs as a competitor to block the binding of biotinylated mAbs [See Additional file 2]. The binding of biotin-RMT2-14 was blocked by RMT2-14 and RMT2-25, but not by RMT2-26. In contrast, the binding of biotin-RMT2-25 or biotin-RMT2-26 was blocked by RMT2-25 and RMT2-26, but not by RMT2-14. These results indicated that three mAbs bound to related but different epitopes in the TIM-2 molecule.

A previous report showed that TIM-2 bound to Sema4A [6]. Thus, we generated Sema4A-Ig fusion protein and Sema4A-transfected cells, and examined the binding to TIM-2. However, we could not confirm the binding of Sema4A-Ig to TIM-2-transfected cells or the binding of TIM-2-Ig to Sema4A-transfected cells by flow cytometry (data not shown). Another report revealed that TIM-2 bound to H-ferritin [4]. To examine whether H-ferritin can bind to TIM-2/NRK cells, we prepared an Alexa647-labeled human recombinant H-ferritin. As shown in Figure 1b, Alexa647-labeled H-ferritin bound to TIM-2/NRK cells, but not to parental NRK or the other TIM family-transfected NRK cells. Moreover, preincubation with our anti-TIM-2 mAbs blocked the H-ferritin binding to TIM-2/L5178Y cells (Figure 1c). RMT2-25 and RMT2-26 showed somewhat stronger blocking activities than RMT2-14.

### Anti-TIM-2 mAb treatment exacerbates CIA

**Figure 2:**
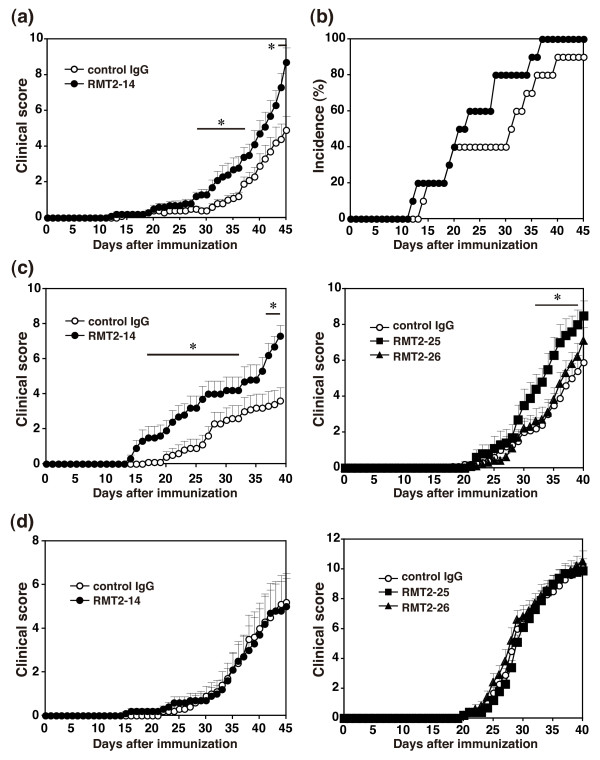
**Effect of anti-TIM-2 mAbs at different phases of CIA**. **(a-b) **Exacerbation of collagen-induced arthritis (CIA) by RMT2-14 treatment. DBA/1 mice were immunized with primary type II collagen (CII)/complete Freund's adjuvant (CFA) on day 0 and secondary CII/incomplete Freund's adjuvant (IFA) on day 21. Two groups of mice were treated with RMT2-14 or control IgG every three days from day 0 to day 42. **(a) **Clinical score and **(b) **incidence of arthritis were evaluated from day 0. **(c) **Effect of anti-T cell immunoglobulin and mucin domain (TIM)-2 monoclonal antibodies (mAbs) at the early phase of CIA. Mice were immunized with CII/CFA once on day 0 and treated with RMT2-14, RMT2-25, RMT2-26, or control IgG from day 0 to day 17. Clinical score of arthritis was evaluated from day 0. **(d) **Effect of anti-TIM-2 mAbs at the late phase of CIA. Mice were immunized with CII/CFA once on day 0 and treated with RMT2-14, RMT2-25, RMT2-26, or control IgG from day 15 to day 32. Clinical score of arthritis was evaluated from day 0. Results are presented as the mean ± standard error of the mean of 10 mice in each group. *, *P *< 0.05 as compared with control IgG. Similar results were obtained in three independent experiments.

**Figure 3:**
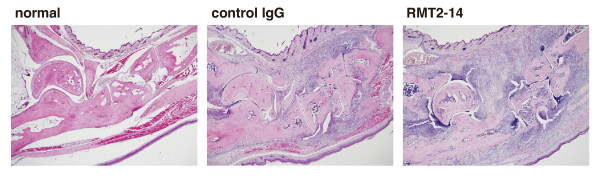
**Effect of anti-TIM-2 mAbs on histopathological arthritis**. Hind paws from normal mice and control IgG- or RMT2-14-treated collagen-induced arthritis (CIA) mice at day 45 were stained with H&E. Original magnification, × 4. Representatives in each group of 10 mice are shown.

To explore the contribution of TIM-2 to the development of autoimmune arthritis, we first administrated anti-TIM-2 mAb (RMT2-14) or control IgG from day 0 to day 42 into the CIA mice. DBA/1 mice were immunized with CII/CFA on day 0 and with CII/IFA on day 21. As shown in Figure 2a, clinical score of arthritis was assessed from the day 0. When mice were treated with RMT2-14, clinical score was significantly more severe than the control IgG-treated mice (*P *< 0.05 on day 28 to 37 and day 44 to 45). Additionally, the incidence of disease was higher in the RMT2-14-treated group than control IgG-treated group (Figure 2b, P = 0.183). Histological analysis of the joints also showed more severe arthritis in the RMT2-14-treated mice compared with the control IgG-treated mice (Figure 3). The hind paw sections from RMT-2-14-treated mice showed more extensive infiltration of mononuclear cells, synovial hyperplasia, pannus formation, and cartilage destruction as compared with the control IgG-treated mice. These results suggested a substantial contribution of TIM-2 to the pathogenesis of CIA.

### Effect of anti-TIM-2 mAbs during early or late phase of CIA

We next examined the effect of anti-TIM-2 mAbs during the early phase or the late phase of CIA. Mice were immunized with CII/CFA only once on day 0 and treated with anti-TIM-2 mAbs (RMT2-14, RMT2-25, or RMT2-26) or control IgG from day 0 to day 17 for the early phase or from day 15 to day 32 for the late phase. In the early phase, administration of RMT2-14 (*P *< 0.05 on day 17 to 27 and day 37 to 39) and RMT2-25 (*P *< 0.05 on day 32 to 39) significantly enhanced the development of CIA as compared with control IgG (Figure 2c). In contrast, administration of RMT2-26 did not affect the development of CIA (Figure 2c). In the late phase, administration of RMT2-14, RMT2-25, or RMT2-26 did not affect the disease severity (Figure 2d). These results indicate that the exacerbation of arthritis by RMT2-14 and RMT2-25 is implicated in the early phase of CIA development.

### Effect of anti-TIM-2 mAb treatment on the development of antigen-specific T cells

**Figure 4:**
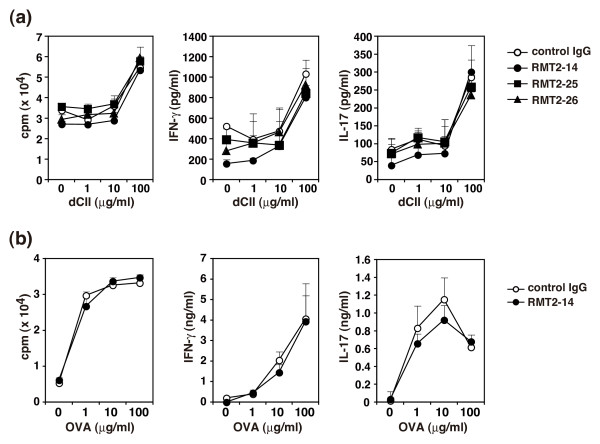
**Effect of anti-TIM-2 mAb treatment on antigen-specific T cell proliferation and cytokine production**. **(a) **DBA/1 mice were immunized with type II collagen (CII)/complete Freund's adjuvant (CFA) on day 0 and CII/incomplete Freund's adjuvant (IFA) on day 21 and treated with RMT2-14, RMT2-25, RMT2-26, or control IgG from day 0 to day 42. Draining lymph node (LN) cells from 19 mice were isolated and pooled in each group at day 45 and cultured with the indicated concentrations of denatured CII (dCII). For estimating proliferation, 0.5 μCi ^3^H-thymidine was added during the last eight hours of a 96-hour culture. Production of IFN-γ and IL-17 in the culture supernatants at 120 hours was determined by ELISA. **(b) **DBA/1 mice were immunized with ovalbumin (OVA)/CFA on day 0 and treated with RMT2-14 or control IgG on days 0, 2, and 4. Draining LN cells from five mice were isolated and pooled in each group on day 7 and cultured with the indicated concentrations of OVA. For estimating proliferation, 0.5 μCi ^3^H-thymidine was added during the last six hours of a 72-hour culture. Production of IFN-γ and IL-17 in the culture supernatants at 72 hours was determined by ELISA. Results are expressed as the mean ± standard deviation of triplicate samples. Similar results were obtained in three independent experiments.

The exacerbation of arthritis by anti-TIM-2 mAbs might result from modulation of CII-specific CD4 T cell responses. To address this possibility, DBA/1 mice were immunized with CII/CFA on day 0 and CII/IFA on day 21 and treated with RMT2-14, RMT2-25, RMT2-26, or control IgG from day 0 to day 42. Draining LN cells were isolated at day 45, and proliferative response and Th1 and Th17 cytokine production (IFN-γ and IL-17) against dCII were assessed. As shown in Figure 4a, dCII-specific proliferative response and production of IFN-γ and IL-17 were almost comparable between the anti-TIM-2 mAb-treated mice and the control IgG-treated mice (*P*>0.05 at every concentration of dCII amongst every group). IL-4 and IL-5 were also measured but not detectable in the culture supernatants (data not shown).

To further evaluate the effect of anti-TIM-2 mAb on the early phase of CII-specific Th1 and Th17 cells, DBA/1 mice were immunized with CII/CFA on day 0 and treated with RMT2-14 or control IgG every three days from day 0 to day 12. LN cells were isolated at day 14, and proliferative response and cytokine production against dCII were assessed [See Additional file 3]. However, both proliferative response and cytokine production (IFN-γ and IL-17) were also comparable between the RMT2-14-treated mice and the control IgG-treated mice (*P*>0.05 at every concentration of dCII).

To further evaluate in the effect of anti-TIM-2 mAbs on the priming of antigen-specific CD4 T cells, DBA/1 mice were immunized with OVA/CFA and treated with RMT2-14 or control IgG on days 0, 2, and 4. LN cells were harvested on day 7, restimulated *in vitro *with various doses of OVA, and proliferative response and cytokine production (IFN-γ and IL-17) were assessed. As shown in Figure 4b, neither proliferative response nor cytokine production were affected by the RMT2-14 treatment as compared with the control IgG treatment (*P*>0.05 at every concentration of dCII). IL-4 and IL-5 were measured but not detectable (data not shown). Taken together, these results suggest that the anti-TIM-2 mAbs treatment do not affect the development/induction of Th1 and Th17 cells, particularly the priming of Th1 and Th17 responses.

### Expression of TIM-2 on B cells but not CD4 T cells

**Figure 5:**
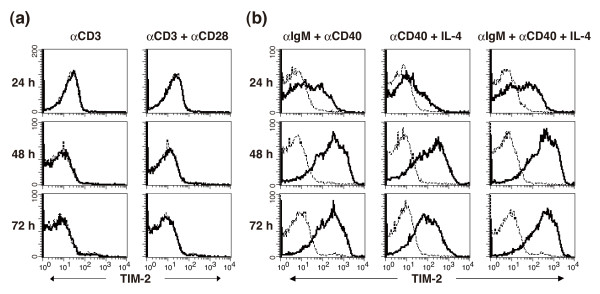
**Expression of TIM-2 on CD4 T and B cells**. **(a) **Expression of T cell immunoglobulin and mucin domain (TIM)-2 on activated CD4 T cells. Purified splenic CD4 T cells were stimulated by immobilized anti-CD3 monoclonal antibody (mAb) with or without anti-CD28 mAb and harvested at the indicated periods. Cells were stained with biotinylated RMT2-26 or control IgG followed by PE-labeled streptavidin. **(b) **Expression of TIM-2 on activated B cells. Purified splenic B cells were stimulated with the indicated combinations of anti-IgM Ab, anti-CD40 mAb, and IL-4. Cells were harvested at the indicated periods and stained with biotinylated RMT2-26 or control IgG followed by PE-labeled streptavidin. Thick lines indicate the staining with anti-TIM-2 mAb and the dotted lines indicate background staining with control IgG.

We further examined the expression of TIM-2 on splenic CD4 T cells by flow cytometric analysis using RMT2-26. TIM-2 expression was not detected on freshly isolated splenic CD4 T cells (data not shown). To determine the expression of TIM-2 upon T cell activation, splenic CD4 T cells were stimulated with immobilized anti-CD3 mAb in the presence or absence of soluble anti-CD28 mAb for 24 to 72 hours. As shown in Figure 5a, TIM-2 expression was not found on CD4 T cells even when stimulated with both anti-CD3 and anti-CD28 mAbs. We also examined the expression of TIM-2 on splenic B cells, but TIM-2 expression was not detected on freshly isolated splenic B cells (data not shown). To determine the expression of TIM-2 upon B cell activation, splenic B cells were stimulated with combinations of anti-IgM, anti-CD40, and recombinant IL-4 for 24 to 72 hours. As shown in Figure 5b, the stimulation with anti-IgM + anti-CD40 or anti-CD40 mAb + IL-4 up-regulated TIM-2 expression on B cells. The combination of anti-IgM + anti-CD40 + IL-4 markedly enhanced TIM-2 expression at 48 to 72 hours (Figure 5b). Similar results were obtained when RMT2-14 or RMT2-25 were used for staining (data not shown). Moreover, RMT2-25 and RMT2-26 precipitated the approximately 55 kDa protein, which was also precipitated from TIM-2/L5178Y cells, from B cells stimulated with anti-IgM + anti-CD40 + IL-4 for 48 hours [See Figure S1b in Additional file 1].

### Effect of anti-TIM-2 mAbs treatment on CII-specific antibody production

**Figure 6:**
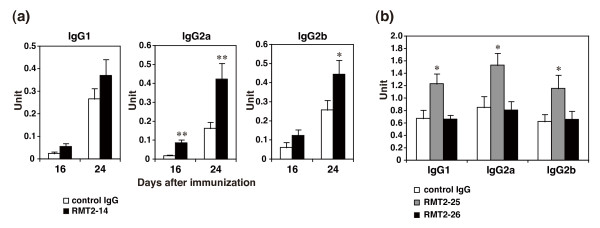
**Effect of anti-TIM-2 mAb treatment on serum anti-CII IgG titers**. **(a) **DBA/1 mice were immunized with type II collagen (CII)/complete Freund's adjuvant (CFA) on day 0 and treated with RMT2-14 or control IgG from day 0 to day 17. Serum levels of anti-CII IgG1, IgG2a, and IgG2b were measured by ELISA on day 16 and 24 after immunization. **(b) **DBA/1 mice were immunized with CII/CFA on day 0 and treated with RMT2-25, RMT2-26, or control IgG from day 0 to day 17. Serum levels of anti-CII IgG1, IgG2a, and IgG2b were measured by ELISA on day 32. Results are expressed as the mean ± standard error of the mean of 10 mice in each group. *, *P *< 0.05; **, *P *< 0.01 as compared with control IgG. TIM, T cell immunoglobulin and mucin domain.

We next investigated the CII-specific IgG1, IgG2a, and IgG2b Ab levels in the sera from the mice, which were immunized with CII/CFA once and treated with anti-TIM-2 mAbs or control IgG in the early phase. As shown in Figure 6a, the serum levels of anti-CII IgG2a (day 16; 0.017 ± 0.006 vs 0.086 ± 0.016 unit, control IgG vs RMT2-14, *n *= 10, *P *< 0.001, day 24; 0.163 ± 0.033 vs 0.423 ± 0.084 unit, control IgG vs RMT2-14, *n *= 10, *P *= 0.004) and IgG2b (day 24; 0.257 ± 0.051 vs 0.444 ± 0.074 unit, control IgG vs RMT2-14, *n *= 10, *P *= 0.044) Abs were significantly increased in the RMT2-14-treated mice as compared with the control-IgG-treated mice. Similarly, the early-phase treatment with RMT2-25 significantly enhanced anti-CII IgG1 (0.675 ± 0.133 vs 1.234 ± 0.16 unit, control IgG vs RMT2-14, *n *= 8, *P *= 0.031), IgG2a (0.853 ± 0.177 vs 1.533 ± 0.191 unit, control IgG vs RMT2-14, *n *= 8, *P *= 0.035), and IgG2b (0.623 ± 0.117 vs 1.159 ± 0.214 unit, control IgG vs RMT2-14, *n *= 8, *P *= 0.049) Abs at day 32 after immunization (Figure 6b). In contrast, RMT2-26 treatment did not affect the CII-specific Ab production (Figure 6b, *n *= 8, *P*>0.8 each isotype). These results raise the possibility that the exacerbation of CIA by RMT2-14 and RMT2-25 resulted from the enhancement of anti-CII Abs production.

### Effect of anti-TIM-2 mAbs on B cell proliferation *in vitro*

**Figure 7:**
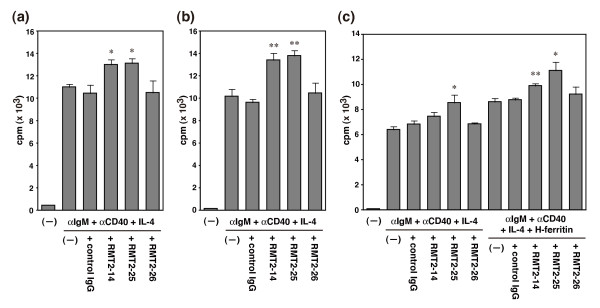
**Effect of anti-TIM-2 mAbs on B cell proliferation *in vitro***. Purified splenic B cells from **(a) **DBA/1, **(b) **BALB/c, or **(c) **FcRγ-deficient mice were stimulated with anti-IgM, anti-CD40, and IL-4 in the absence or presence of H-ferritin and indicated monoclonal antibodies (mAbs) for 48 hours. Proliferative response was assessed by pulsing the cultures with 0.5 μCi/well ^3^H-thymidine for the last six hours. Data are expressed as the mean ± standard error of the mean of triplicate wells. *, *P *< 0.05; **, *P *< 0.01 as compared with control IgG. The results are representative of two experiments in each mouse strain. TIM, T cell immunoglobulin and mucin domain.

Given the dynamic expression of TIM-2 on B cells, it seems likely that TIM-2 regulates B cell activation or function, and both RMT2-14 and RMT2-25 can block or stimulate B cells by signaling through TIM-2. To address this possibility, splenic B cells from DBA/1 mice were stimulated with anti-IgM, anti-CD40, and IL-4 in the presence of anti-TIM-2 mAbs or control IgG for 48 hours, and then the proliferative response was assessed. As shown in Figure 7a, proliferation of anti-IgM/anti-CD40/IL-4-stimulated B cells was not affected by the addition of control IgG or RMT2-26 (10458.5 ± 725.6 vs 10507.3 ± 1063.7 cpm, control IgG vs RMT2-26, *P *= 0.971). In contrast, the addition of RMT2-14 (10458.5 ± 725.6 vs 13008.3 ± 725.6 cpm, control IgG vs RMT2-14, *P *= 0.025) and RMT2-25 (10458.5 ± 725.6 vs 13129.3 ± 418.4 cpm, control IgG vs RMT2-25, *P *= 0.019) significantly enhanced the proliferation. Similar results were obtained when B cells were purified from the spleen of BALB/c mice (Figure 7b; 9632.8 ± 293.6 control IgG, vs 10465.8 ± 911.3 RMT2-26, *P *= 0.418, vs 13420 ± 615.5 RMT2-14, *P *<0.001, vs 13811.8 ± 459.3 RMT2-25, *P *< 0.001). To avoid positive or negative signaling through FcγRs [15], B cells were purified from FcRγ-deficient mice and anti-CD16/32 mAb was added into the culture. As shown in Figure 7c, proliferation of anti-IgM/anti-CD40/IL-4-stimulated B cells not affected by the addition of control IgG or RMT2-26 (6844.8 ± 272.7 vs 6861 ± 112 cpm, control IgG vs RMT2-26, *P *= 0.958). In contrast, the addition of RMT2-25 significantly enhanced the proliferation (6844.8 ± 272.7 vs 8566.3 ± 616.2 cpm, control IgG vs RMT2-25, *P *= 0.43). The addition of RMT2-14 slightly enhanced the proliferation, but not significant (6844.8 ± 272.7 vs 7459.5 ± 329.1 cpm, control IgG vs RMT2-14, *P *= 0.2). In addition, the proliferation of B cells was also enhanced by the addition of H-ferritin (Figure 7c, P < 0.01). Unexpectedly, the addition of RMT2-14 (8788 ± 160 vs 9907.5 ± 172.9 cpm, control IgG vs RMT2-14, *P *= 0.003) or RMT2-25 (8788 ± 160 vs 11127.5 ± 664.6 cpm, control IgG vs RMT2-25, *P *= 0.014) further enhanced the B cell proliferation rather than to block the enhancement by H-ferritin. In contrast, RMT2-26 did not affect this (8788 ± 160 vs 9227.8 ± 595.9 cpm, control IgG vs RMT2-26, *P *= 0.5). We further examined that naive splenic B cells from DBA/1 mice were stimulated with anti-IgM, anti-CD40, and IL-4 in the presence of anti-TIM-2 mAbs or control IgG, and H-ferritin was subsequently added to the culture after 24 hours. The addition of RMT2-14 and RMT2-25 significantly enhanced the proliferation [See Additional file 4].

### RMT2-14 and RMT2-25 enhances production of IgG2b and IgG3 *in vitro*

**Figure 8:**
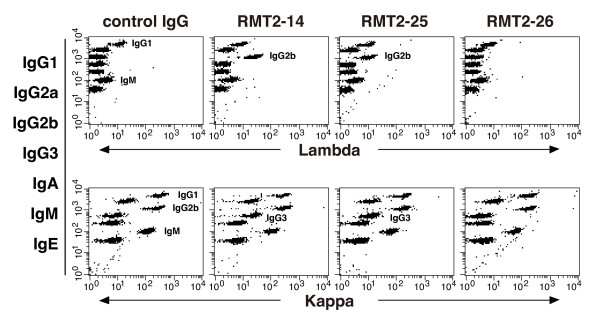
**Effect of anti-TIM-2 mAbs on Ig production *in vitro***. Purified B cells from FcRγ-deficient mice were stimulated with anti-IgM, anti-CD40, and IL-4 in the presence of the indicated anti-T cell immunoglobulin and mucin domain (TIM)-2 monoclonal antibodies (mAbs) or control IgG. Culture supernatants were harvested at day 7 and analyzed using a CBA assay that could identify all seven mouse Ig isotypes in a single sample. Presence of a particular isotype is indicated by lambda (upper) and kappa (lower) fluorescence (with labels provided to indicate positive cultures). Data are representative of three experiments.

We further examined whether anti-TIM-2 stimulation on B cells influenced secretion of each Ig isotype. Purified splenic B cells were stimulated with anti-IgM, anti-CD40, and IL-4 in the presence of anti-TIM-2 mAbs or control rat IgG. Culture supernatants were harvested at day 7 and subjected to a CBA assay for the seven mouse Ig isotypes [16,17]. As shown in Figure 8, supernatants from the RMT2-14 and RMT2-25 cultures showed higher IgG2b/λ and IgG3/κ levels as compared with the control IgG and RMT-2-26 cultures. Collectively, these results suggest that the exacerbation of CIA by RMT2-14 and RMT2-25 was caused by enhancement of B cell activation and Ab production through agonistic stimulation of TIM-2 on B cells by these mAbs.

## Discussion

To explore the contribution of TIM-2 to the development of CIA, we generated anti-mouse TIM-2 mAbs (RMT2-14, RMT2-25, and RMT2-26), which bound to TIM-2 cDNA transfectants but not to those expressing the other TIM family molecules (TIM-1 B6, TIM-1 BALB, TIM-3 B6, TIM-3 BALB, and TIM-4). All three mAbs precipitated an approximately 55 kDa protein from TIM-2/L5178Y cells, consistent with the molecular mass of TIM-2 previously reported [6]. Moreover, all of these mAbs inhibited the binding of H-ferritin to TIM-2 transfectants. These results indicate that the anti-TIM-2 mAbs used in this study are specific for mouse TIM-2 and can interrupt the interaction between TIM-2 and H-ferritin. RMT2-14- or RMT2-25-treated mice showed a substantially enhanced development of CIA. Moreover, the administration of RMT2-14 or RMT2-25 during the early phase effectively exacerbated the disease severity, although it was not effective during the late phase. *In vitro *restimulation of draining LN cells showed that the anti-TIM-2 mAb treatment did not affect dCII-specific proliferative response or production of Th1 and Th17 cytokines (IFN-γ and IL-17). These results suggest that TIM-2 dose not play a major role in the development of Th1 or Th17 cells during the early phase. In support of this theory, we also found that the anti-TIM-2 mAb treatment did not inhibit the priming of OVA-reactive Th1 and Th17 cells induced by OVA/CFA immunization. On the other hand, it was notable that the treatment with RMT2-14 or RMT2-25 enhanced the serum levels of anti-CII IgG1, IgG2a, and IgG2b Abs. Moreover, RMT2-14 or RMT2-25 enhanced B cell proliferation and Ig production *in vitro*. Collectively, these results suggest that TIM-2 delivers a signal into B cells, which enhances proliferation and Ab production and that the exacerbation of CIA by RMT2-14 and RMT2-25 resulted from the enhancement of B cell activation and function by agonistic effects of these mAbs.

Several recent studies have indicated that TIM-2 may have inhibitory functions in Th2 immune responses. TIM-2-Ig fusion protein induced T cell hyperproliferation and enhanced production of Th2 cytokines *in vivo *[9]. A subsequent study also showed that TIM-2-Ig-treated or TIM-2-deficient mice showed exacerbated lung inflammation in the OVA-induced asthma model [10]. These results suggested that TIM-2 could be involved in the suppression of Th2-mediated immune responses. TIM-2 was not expressed on CD4 T cells constitutively but up-regulated following activation for Th2 condition but not Th1 condition [9]. We show here that TIM-2 expression was not found on primary activated CD4 T cells and the anti-TIM-2 mAb treatment did not affect Th1 and Th17 responses, suggesting that TIM-2 signaling dose not contribute to the development of Th1 or Th17 cells in CIA. Thus, the present enhancement of arthritis severity by anti-TIM-2 mAbs does not result from inhibition of Th2 response or an augmentation of Th1 or Th17 response. However, a previous study has indicated that the administration of TIM-2-Ig at the induction phase or just before disease onset reduced the severity of experimental autoimmune encephalomyelitis (EAE), which is a Th17-mediated autoimmune disease model [9]. The mechanism for such differential roles of TIM-2 in EAE above and CIA is presently unknown. It is possible that TIM-2 has some additional binding molecule other than H-ferritin and Sema4A. TIM-2-Ig, but not anti-TIM-2 mAbs, may interrupt the interaction between TIM-2 and the unknown ligand or receptor. Further studies are required to address this possibility.

A previous study by Chen et al. indicated that Sema4A-Ig fusion protein does not bind to the TIM-2-transfectants [4]. Consistent with this report, we have been unable to confirm the binding of Sema4A and TIM-2 (data not shown). On the other hand, we observed that H-ferritin bound to mouse TIM-2-transfected cells. A previous study has suggested that H-ferritin can impair the maturation of B cells *in vitro *[18]. In our present study, H-ferritin enhanced B cell proliferation induced by anti-IgM, anti-CD40, and IL-4. This enhancement was not blocked by anti-TIM-2 mAbs, whereas these mAbs could inhibit the binding of H-ferritin to TIM-2 transfectants. Notably, RMT2-14 and RMT2-25 did not inhibit H-ferritin binding to activated B cells [See Additional file 5]. Therefore, some molecules other than TIM-2 might be responsible for the enhancement of B cell proliferation by H-ferritin. In any case, H-ferritin-TIM-2 interaction might not contribute to the development of CIA, because the administration of RMT2-26, which could block the H-ferritin-TIM-2 interaction as efficiently as RMT2-25 and more efficiently than RMT2-14, did not affect the development of CIA.

It has been demonstrated that the TIM-2 is expressed at low levels on splenic B cells and at higher levels on germinal center B cells [4]. TIM-2 expression was markedly up-regulated on splenic B cells by stimulation with anti-IgM, anti-CD40, and IL-4 *in vitro*. RMT2-14 and RMT2-25 enhanced B cell proliferation and Ig production *in vitro *and anti-CII Ab production *in vivo*. B cells receive multiple signals during their differentiation into antibody-secreting cells. These include signals delivered by antigen through the B-cell receptor that drive proliferation, signals delivered by cytokines that initiate Ig class switching, and signals through CD40 or Toll-like receptors, which synergize with cytokine signals to cause class-switch recombination and antibody secretion. Class-switch recombination involves activation of the gene for activation-induced cytidine deaminase, followed by deletional switch recombination and expression of mature transcripts of the switched Ig isotype [19-21]. To confirm the contribution of TIM-2 signal to the induction of class-switch recombination, therefore, further studies are required whether TIM-2 signaling can induce activation of activation-induced cytidine deaminase.

## Conclusions

The administration of anti-TIM-2 mAbs exacerbated CIA through enhancement of B cell activation and Ab production during the early phase, but not induction of Th1 or Th17 cells. Mouse TIM-2 and TIM-1 are highly homologous, but it is unclear whether they have similar functions. It has been suggested that mouse TIM-2 may share some functions with human TIM-1 based on a close sequence homology. We have observed that mouse TIM-1 is also highly expressed on splenic B cells after stimulation with anti-IgM, anti-CD40, and IL-4 *in vitro *(unpublished observation). Therefore, it is possible that human TIM-1 may contribute to B cell activation and Ab production in rheumatoid arthritis patients, as revealed for mouse TIM-2 in CIA mice.

## Abbreviations

Ab: antibody; BSA: bovine serum albumin; CBA: cytometric bead array; CFA: complete Freund's adjuvant; CIA: collagen-induced arthritis; CII: type II collagen; dCII: denatured CII; ELISA: enzyme-linked immunosorbent assay; H-ferritin: the heavy chain of ferritin; IFA: incomplete Freund's adjuvant; IFN: interferon; IL: interleukin; LN: lymph node; mAb: monoclonal antibody; OVA: ovalbumin; PBS: phosphate-buffered saline; RT-PCR: reverse transcription polymerase chain reaction; Sema4A: semaphoring 4A; Th: T helper; TIM: T cell immunoglobulin and mucin domain.

## Competing interests

The authors declare that they have no competing interests.

## Authors' contributions

TK and YA contributed to the design, acquisition and interpretation of data. JI and FM assisted with the experiments. YK performed the histological experiments. YU and JM generated anti-TIM2 mAbs. SM participated in the design of the study. HY, KO, and YK contributed to the planning of the research. HA contributed to the conception and design of the study, data analysis and interpretation, and manuscript writing. All authors read and approved the final manuscript.

## Supplementary Material

Additional file 1 **Immunoprecipitation of TIM-2 antigen with anti-TIM-2 mAbs**. **(a) **T cell immunoglobulin and mucin domain (TIM)-2/L5178Y or L5178Y cells (1 × 10^7^) were lysed in a lysis buffer containing 0.5% Nonidet P-40, 50 mM Tris, and 250 mM NaCl. **(b) **Purified splenic B cells (1 × 10^7^) from DBA/1 mice were stimulated with anti-IgM, anti-CD40, and IL-4 for 48 hours and lysed in the lysis buffer. The cleared lysates were immunoprecipitated with RMT2-14-, RMT2-25-, RMT2-26-, rat IgG2a-, or rat IgG2b-preloaded protein G-Sepharose. The beads were washed with the lysis buffer, and bound proteins were eluted with 1% SDS sample buffer, subjected to 10% SDS-PAGE under nonreducing condition, and then blotted onto polyvinylidene difluoride membrane (Millipore). The blotted proteins were detected using biotin-conjugated RMT2-14, RMT2-25, RMT2-26, rat IgG2a, or rat IgG2b followed by avidin-biotinylated peroxidase complex and SuperSignal West Dure Extended Duration Substrate. The positions of molecular mass markers are indicated at the right in kilodaltons. Click here for file

Additional file 2 **Competitive inhibition test**. T cell immunoglobulin and mucin domain (TIM)-2/L5178Y cells were pre-incubated with 10 μg of RMT2-14, RMT2-25, RMT-2-26, or control rat IgG and then stained with biotinylated RMT2-14 (0.5 μg), RMT2-25 (0.1 μg), or RMT-2-26 (0.1 μg) followed by PE-labeled streptavidin to determine whether these monoclonal antibodies (mAbs) recognized different TIM-2 antigen epitopes. Thick lines indicate the staining with the respective mAb and the dotted lines indicate background staining with control IgG. Click here for file

Additional file 3 **Effect of anti-TIM-2 mAb treatment on antigen-specific T cell proliferation and cytokine production**. DBA/1 mice were immunized with type II collagen (CII)/complete Freund's adjuvant (CFA) on day 0 and treated with RMT2-14 or control IgG every three days from day 0 to day 12. Draining lymph node (LN) cells from 10 mice were isolated and pooled at day 14 and cultured with the indicated concentrations of denatured CII (dCII). For estimating proliferation, 0.5 μCi 3H-thymidine was added during the last six hours of a 96-hour culture. Production of IFN-γ and IL-17 in the culture supernatants at 120 hour was determined by ELISA. IL-4 and IL-5 were not detectable in the culture supernatants. Results are expressed as the mean ± standard deviation. Click here for file

Additional file 4 **Effect of anti-TIM-2 mAbs on B cell proliferation *in vitro***. Purified small resting splenic B cells from DBA/1 mice were stimulated with anti-IgM, anti-CD40, and IL-4 in the presence of anti-T cell immunoglobulin and mucin domain (TIM)-2 monoclonal antibodies (mAbs) or control IgG, and H-ferritin was added to the culture after 24 hours. Proliferative response was assessed by pulsing the cultures with 0.5 μCi/well ^3^H-thymidine for the last six hours of 72 hours. Data are expressed as the mean ± standard error of the mean of triplicate wells. *, *P *< 0.05 as compared with control IgG. Click here for file

Additional file 5 **Anti-TIM-2 mAbs do not inhibit H-ferritin binding to activated B cells**. Purified splenic B cells were stimulated with the combination of anti-IgM, anti-CD40 monoclonal antibody (mAb), and IL-4 for 72 hours. Cells were pre-incubated with 10 μg of RMT2-14, RMT2-25, or control rat IgG and then stained with Alexa647-labeled H-ferritin. Thick lines indicate the staining with Alexa647-labeled H-ferritin and the dotted lines indicate background staining with PBS. Click here for file
