# Supplementary material for: Anti-T cell immunoglobulin and mucin domain-2 monoclonal antibody exacerbates collagen-induced arthritis by stimulating B cells
Source: Arthritis Res Ther. 2011 Mar 22;13(2):R47. doi: 10.1186/ar3288 (PMC3132034; doi:10.1186/ar3288)
Supplement: Additional file 1 — Immunoprecipitation of TIM-2 antigen with anti-TIM-2 mAbs. (a) T cell immunoglobulin and mucin domain (TIM)-2/L5178Y or L5178Y cells (1 × 10^7^) were lysed in a lysis buffer containing 0.5% Nonidet P-40, 50 mM Tris, and 250 mM NaCl. (b) Purified splenic B cells (1 × 10^7^) from DBA/1 mice were stimulated with anti-IgM, anti-CD40, and IL-4 for 48 hours and lysed in the lysis buffer. The cleared lysates were immunoprecipitated with RMT2-14-, RMT2-25-, RMT2-26-, rat IgG2a-, or rat IgG2b-preloaded protein G-Sepharose. The beads were washed with the lysis buffer, and bound proteins were eluted with 1% SDS sample buffer, subjected to 10% SDS-PAGE under nonreducing condition, and then blotted onto polyvinylidene difluoride membrane (Millipore). The blotted proteins were detected using biotin-conjugated RMT2-14, RMT2-25, RMT2-26, rat IgG2a, or rat IgG2b followed by avidin-biotinylated peroxidase complex and SuperSignal West Dure Extended Duration Substrate. The positions of molecular mass markers are indicated at the right in kilodaltons. [file ar3288-S1.PDF]

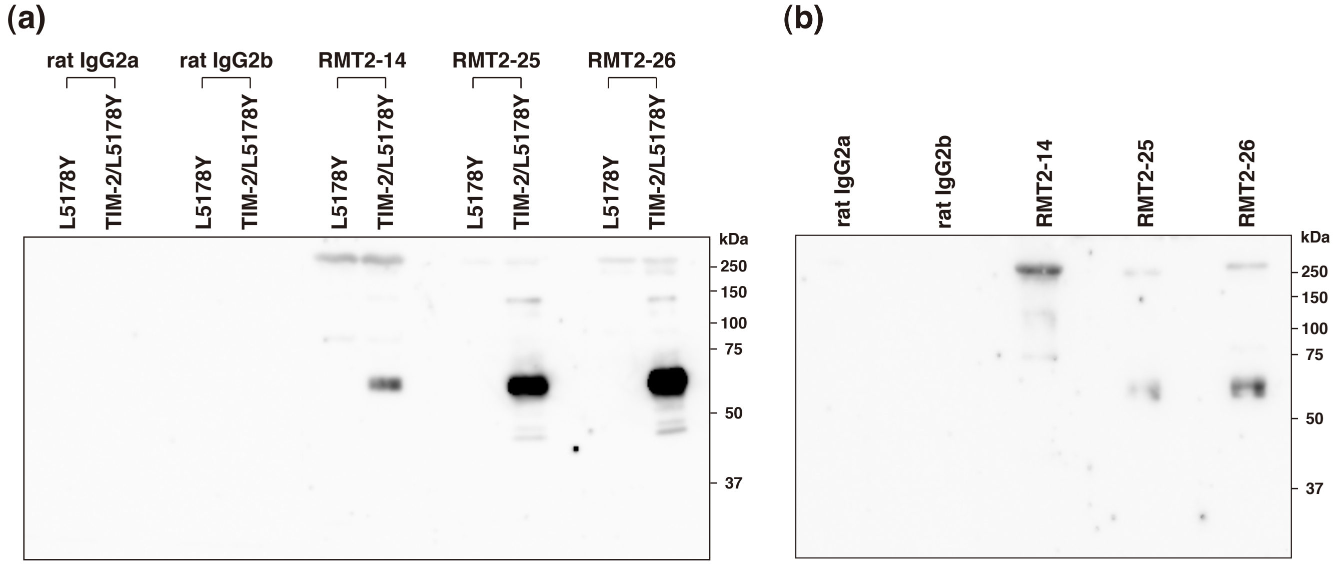

Figure S1 Immunoprecipitation of TIM-2 antigen with anti-TIM-2 mAbs.

(a) TIM-2/L5178Y or L5178Y cells (1 x 10^7) were lysed in a lysis buffer containing 0.5% Nonidet P-40, 50 mM Tris, and 250 mM NaCl. **(b)** Purified splenic B cells (1 x 10^7) from DBA/1 mice were stimulated with anti-IgM, anti-CD40, and IL-4 for 48 h and lysed in the lysis buffer. The cleared lysates were immunoprecipitated with RMT2-14-, RMT2-25-, RMT2-26-, rat IgG2a-, or rat IgG2b-preloaded protein G-Sepharose. The beads were washed with the lysis buffer, and bound proteins were eluted with 1% SDS sample buffer, subjected to 10% SDS-PAGE under nonreducing condition, and then blotted onto polyvinylidene difluoride membrane (Millipore). The blotted proteins were detected using biotin-conjugated RMT2-14, RMT2-25, RMT2-26, rat IgG2a, or rat IgG2b followed by avidin-biotinylated peroxidase complex (Vector Laboratories) and SuperSignal West Dure Extended Duration Substrate (Thermo Fisher Scientific). The positions of molecular mass markers are indicated at the right in kilodaltons.
