# Supplementary material for: Anti-T cell immunoglobulin and mucin domain-2 monoclonal antibody exacerbates collagen-induced arthritis by stimulating B cells
Source: Arthritis Res Ther. 2011 Mar 22;13(2):R47. doi: 10.1186/ar3288 (PMC3132034; doi:10.1186/ar3288)
Supplement: Additional file 2 — Competitive inhibition test. T cell immunoglobulin and mucin domain (TIM)-2/L5178Y cells were pre-incubated with 10 μg of RMT2-14, RMT2-25, RMT-2-26, or control rat IgG and then stained with biotinylated RMT2-14 (0.5 μg), RMT2-25 (0.1 μg), or RMT-2-26 (0.1 μg) followed by PE-labeled streptavidin to determine whether these monoclonal antibodies (mAbs) recognized different TIM-2 antigen epitopes. Thick lines indicate the staining with the respective mAb and the dotted lines indicate background staining with control IgG. [file ar3288-S2.PDF]

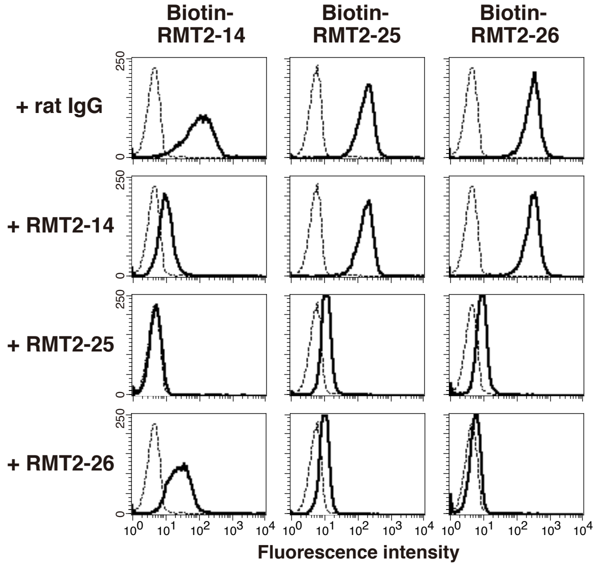

Figure S2 Competitive inhibition test.

TIM-2/L5178Y cells were pre-incubated with 10 μg of RMT2-14, RMT2-25, RMT-2-26, or control rat IgG and then stained with biotinylated RMT2-14 (0.5 μg), RMT2-25 (0.1 μg), or RMT-2-26 (0.1 μg) followed by PE-labeled streptavidin to determine whether these mAbs recognized different TIM-2 antigen epitopes. Thick lines indicate the staining with the respective mAb and the dotted lines indicate background staining with control IgG.
