# Supplementary material for: Anti-T cell immunoglobulin and mucin domain-2 monoclonal antibody exacerbates collagen-induced arthritis by stimulating B cells
Source: Arthritis Res Ther. 2011 Mar 22;13(2):R47. doi: 10.1186/ar3288 (PMC3132034; doi:10.1186/ar3288)
Supplement: Additional file 3 — Effect of anti-TIM-2 mAb treatment on antigen-specific T cell proliferation and cytokine production. DBA/1 mice were immunized with type II collagen (CII)/complete Freund's adjuvant (CFA) on day 0 and treated with RMT2-14 or control IgG every three days from day 0 to day 12. Draining lymph node (LN) cells from 10 mice were isolated and pooled at day 14 and cultured with the indicated concentrations of denatured CII (dCII). For estimating proliferation, 0.5 μCi 3H-thymidine was added during the last six hours of a 96-hour culture. Production of IFN-γ and IL-17 in the culture supernatants at 120 hour was determined by ELISA. IL-4 and IL-5 were not detectable in the culture supernatants. Results are expressed as the mean ± standard deviation. [file ar3288-S3.PDF]

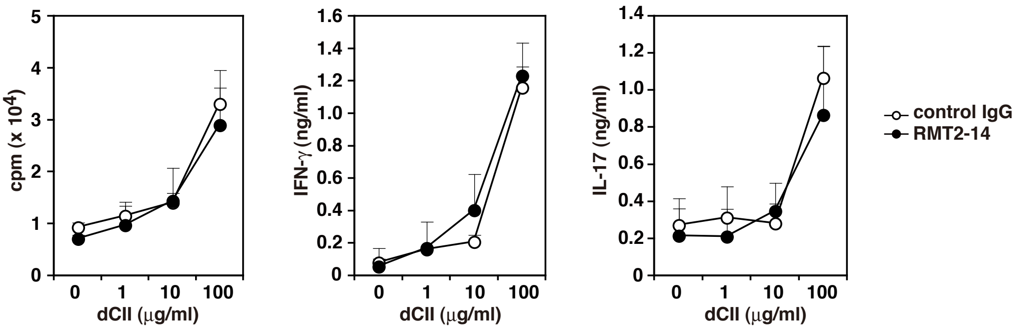

Figure S3 Effect of anti-TIM-2 mAb treatment on antigen-specific T cell proliferation and cytokine production.

DBA/1 mice were immunized with CII/CFA on day 0 and treated with RMT2-14 or control IgG every 3 days from day 0 to day 12. Draining LN cells from ten mice were isolated and pooled at day 14 and cultured with the indicated concentrations of denatured CII (dCII). For estimating proliferation, 0.5 μCi 3H-thymidine was added during the last 6 h of a 96-h culture. Production of IFN-γ and IL-17 in the culture supernatants at 120 h was determined by ELISA. IL-4 and IL-5 were not detectable in the culture supernatants. Results are expressed as the mean ± SD.
