# Supplementary material for: Anti-T cell immunoglobulin and mucin domain-2 monoclonal antibody exacerbates collagen-induced arthritis by stimulating B cells
Source: Arthritis Res Ther. 2011 Mar 22;13(2):R47. doi: 10.1186/ar3288 (PMC3132034; doi:10.1186/ar3288)
Supplement: Additional file 4 — Effect of anti-TIM-2 mAbs on B cell proliferation in vitro. Purified small resting splenic B cells from DBA/1 mice were stimulated with anti-IgM, anti-CD40, and IL-4 in the presence of anti-T cell immunoglobulin and mucin domain (TIM)-2 monoclonal antibodies (mAbs) or control IgG, and H-ferritin was added to the culture after 24 hours. Proliferative response was assessed by pulsing the cultures with 0.5 μCi/well 3H-thymidine for the last six hours of 72 hours. Data are expressed as the mean ± standard error of the mean of triplicate wells. *, P < 0.05 as compared with control IgG. [file ar3288-S4.PDF]

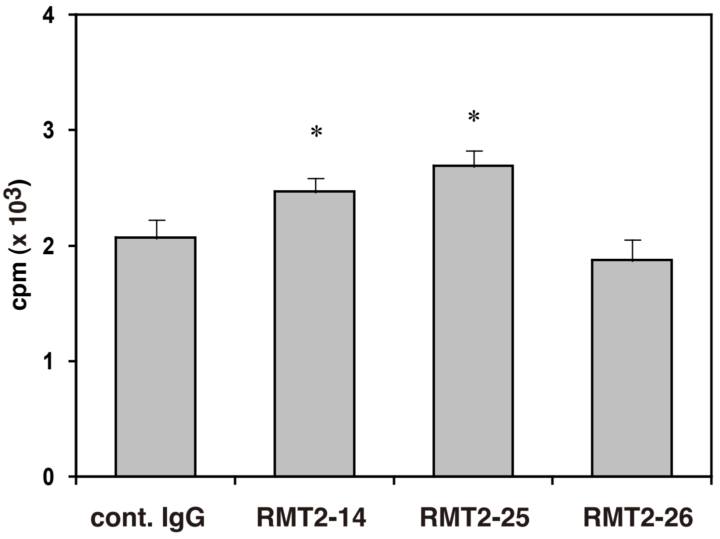

Figure S4 Effect of anti-TIM-2 mAbs on B cell proliferation in vitro.
Purified small resting splenic B cells from DBA/1 mice were stimulated with anti-IgM, anti-CD40, and IL-4 in the presence of anti-TIM-2 mAbs or control IgG, and H-ferritin was added to the culture after 24 h. Proliferative response was assessed by pulsing the cultures with 0.5 μCi/well 3H-thymidine for the last 6 h of 72 h. Data are expressed as the mean ± SEM of triplicate wells. *, $p < 0.05$ as compared to control IgG.
