# Supplementary material for: Anti-T cell immunoglobulin and mucin domain-2 monoclonal antibody exacerbates collagen-induced arthritis by stimulating B cells
Source: Arthritis Res Ther. 2011 Mar 22;13(2):R47. doi: 10.1186/ar3288 (PMC3132034; doi:10.1186/ar3288)
Supplement: Additional file 5 — Anti-TIM-2 mAbs do not inhibit H-ferritin binding to activated B cells. Purified splenic B cells were stimulated with the combination of anti-IgM, anti-CD40 monoclonal antibody (mAb), and IL-4 for 72 hours. Cells were pre-incubated with 10 μg of RMT2-14, RMT2-25, or control rat IgG and then stained with Alexa647-labeled H-ferritin. Thick lines indicate the staining with Alexa647-labeled H-ferritin and the dotted lines indicate background staining with PBS. [file ar3288-S5.PDF]

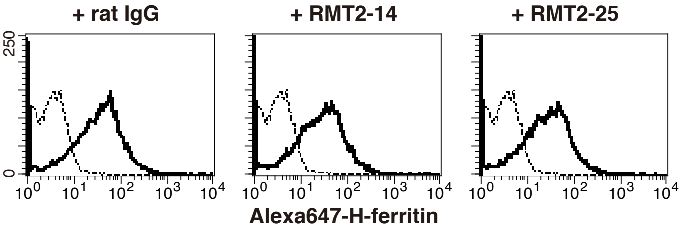

Figure S5 Anti-TIM-2 mAbs do not inhibit H-ferritin binding to activated B cells.
Purified splenic B cells were stimulated with the combination of anti-IgM, anti-CD40 mAb, and IL-4 for 72 h. Cells were pre-incubated with 10 μg of RMT2-14, RMT2-25, or control rat IgG and then stained with Alexa647-labeled H-ferritin. Thick lines indicate the staining with Alexa647-labeled H-ferritin and the dotted lines indicate background staining with PBS.
